# Tea Polysaccharides and Their Bioactivities

**DOI:** 10.3390/molecules21111449

**Published:** 2016-10-30

**Authors:** Ling-Ling Du, Qiu-Yue Fu, Li-Ping Xiang, Xin-Qiang Zheng, Jian-Liang Lu, Jian-Hui Ye, Qing-Sheng Li, Curt Anthony Polito, Yue-Rong Liang

**Affiliations:** 1 Tea Research Institute, Zhejiang University, # 866 Yuhangtang Road, Hangzhou 310058, China; lingling8852@126.com (L.-L.D.); 21516103@zju.edu.cn (Q.-Y.F.); xqzheng@zju.edu.cn (X.-Q.Z.); jllu@zju.edu.cn (J.-L.L.); jianhuiye@zju.edu.cn (J.-H.Y.); qsli@zju.edu.cn (Q.-S.L.); curtpolito@outlook.com (C.A.P.); 2 National Tea and Tea product Quality Supervision and Inspection Center (Guizhou), Zunyi 563100, China; gzzyzj_2009@vip.sina.com

**Keywords:** *Camellia sinensis*, tea polysaccharides, chemical composition, antioxidant, antitumor, antihyperglycemia, anti-inflammation

## Abstract

Tea (*Camellia sinensis*) is a beverage beneficial to health and is also a source for extracting bioactive components such as theanine, tea polyphenols (TPP) and tea polysaccharides (TPS). TPS is a group of heteropolysaccharides bound with proteins. There is evidence showing that TPS not only improves immunity but also has various bioactivities, such as antioxidant, antitumor, antihyperglycemia, and anti-inflammation. However, inconsistent results concerning chemical composition and bioactivity of TPS have been published in recent years. The advances in chemical composition and bioactivities of TPS are reviewed in the present paper. The inconsistent and controversial results regarding composition and bioactivities of TPS are also discussed.

## 1. Introduction

Tea (*Camellia sinensis*) is a beverage widely drunk across the world [1], and its extracts have been used as medicinal and dietary supplements in many countries such as China, Japan and the US [2]. Tea contains a variety of bioactive compounds including tea polyphenols (TPP) [2], theanine [3] and tea polysaccharides (TPS) [4], which contribute to the health benefits of tea. A polysaccharide is a high molecular weight (MW) polymer, consisting of at least ten monosaccharides mutually joined by glycosidic linkages. The glycosyl moiety of the hemiacetal or hemiketal, together with the hydroxyl group of another sugar unit, formed the glycosidic linkages [5]. TPS is a group of heteropolysaccharides extracted from leaves, flowers and seed peels of the tea plant [4]. Great advances have been made in chemical and bioactive studies of TPP or catechins and related tea products over the last few decades. However, TPS has received rare attention. There have been studies showing that TPS has many health benefits including antioxidant, anti-aging, antitumor, antibacterial and anti-skin-aging properties, as well as the ability to inhibit diabetes, improve immunity, and alleviate hepatotoxicity [6,7,8]. The preparation, chemical composition and physiological activities of TPS are reviewed in the present paper.

## 2. Polysaccharides in Tea

### 2.1. Basic Composition of Tea Polysaccharides (TPS)

TPS is a nonstarch protein-bound acidic polysaccharide, which contains 44.2% neutral sugar, 43.1% uronic acid and 3.5% protein [9]. The carbohydrate composition of TPS includes glucose (Glc，128.4 μM), galactose (Gal, 101.4 μM), arabinose (Ara, 71.1 μM), rhamnose (Rha, 47.1 μM), xylose (Xyl, 25.0 μM), galacturonic acid (GalA, 24.0 μM), mannose (Man, 16.3 μM), ribose (Rib, 10.3 μM) and glucuronic acid (GulA, 5.6 μM) [10]. The second-derivative IR spectra of TPS had peak intensity around 1075 cm^−1^ and 1045 cm^−1^, showing TPS characterizes galactopyranose in the backbone and arabinofuranose units in side branches [11].

TPP is a group of abundant bioactive components in tea, and crude TPS usually contains partial TPP. The carbohydrate, protein and polyphenols are conjugated with each other in the crude TPS. The composition of crude TPS varies with processing methods including extraction and drying [12]. Crude TPS1 and TPS2 were obtained when water extracts of green tea were precipitated using 40% and 70% ethanol, respectively. The TPS1 could be further separated on gel permeation into homogeneous water-soluble TPS1-2a and TPS1-2b, which were homogalacturonan (HG) pectins with MW ca. 20 kDa, consisting of a backbone of 1,4-linked GalA residues with 28.4% and 26.1% of carboxyl groups as methyl ester, respectively [13]. The TPS1-2a and TPS1-2b showed a higher phagocytic effect than TPS2.

TPS can be divided into neutral polysaccharides (NTPS) and acid polysaccharides (ATPS). The crude water-soluble TPS could be separated by anion-exchange chromatography into five fractions, i.e., fractions A, B, C, D and E, among which fractions A and C had significant glucokinase-stimulating activity, in which fraction C showed the highest activity and could be further separated by gel filtration chromatography into fractions C-1 and C-2. The FC-1 is an acidic polysaccharide containing 8% GalA but no protein, with MW ca. 60 kDa [14].

Sugars and uronic acids are abundant in TPS. NTPS contains 82.7% total sugar, 12.9% of which is comprised of uronic acid, whereas ATPS contains 85.5% total sugar, 39.8% of which is made up of uronic acid. Sugar composition is mainly Gal (67.6%) in NTPS, but Rha, Ara, Gal and GalA are in ATPS [15]. Nucleic acid was also detected in ATPS [16]. TPS from some tea sources also bear rare earth elements (REE) including La, Ce, and Nd, in which La was more than 75% of total REE. Iron, magnesium, zinc and selenium were also detected in TPS [17].

### 2.2. TPS Variation Between Tea Cultivars and Plant Organs

TPS in leaf cuticular membrane varies with tea cultivar and cell partitions. Tea cultivar “Gokou” has markedly higher TPS than cultivars “Samidori” and “Yabukita” [18]. Among various cell partitions, the adaxial side usually has a higher level of TPS than the abaxial side [18]. Tea leaf TPS (TLPS) is increased with maturity of the tea leaf, with 0.23% in the first leaf and 0.58% in the sixth leaf below apex bud on the same tea shoot [19]. Tea flowers examined in this study contained 5.24% TPS, which is higher than tea leaves (3.64%) [20]. Three kinds of TPS were extracted from tea seeds and the fractions 1, 2 and 3 of tea seed TPS (TSPS) had MW 500 kDa, 130 kDa, and 5 kDa respectively, and they showed typical characteristics of polysaccharides and protein. TSPS mainly consists of Rha, Xyl, Ara, Glc, Gal, GulA and GalA, with a molar ratio of 4.9:1.7:11.1:27.2:14.0:3.4:1. The sugar backbone of TSPS might consist of Glc, but branched chain may comprise Rha, Xyl, Ara and Gal [21]. Tea fruit peel TPS (TFPPS) contained 4.98% of polysaccharides and the TFPPS was a group of acid protein-bound heteropolysaccharides. The major sugars in TFPPS were Rha, Man, Glc, Gal, Ara, Xyl and fucose (Fuc) [22]. Polysaccharides extracted from a hawk mature leaf tea (a herbal tea) (HMPS) were mainly composed of Ara, Gal, Glc and Man and the HMPS can be classified into two fractions, i.e., HMPS-1 with MW 133 kDa and HMPS-2 with MW 100 kDa [23].

### 2.3. Effect of Tea Processing on TPS

Teas can be classified into green tea, black tea, oolong tea and pu-erh tea owing to different processing methods [2]. As early as 1998, two kinds of green tea TPS (GTPS) were separated from green tea infusion, i.e., GTPS-A with MW over 100 kDa and GTPS-B with MW 10 kDa [24]. Crude GTPS was a conjugate consisting of a polysaccharide part and a protein part [25]. GTPS from four green tea sources including “Xihu Longjing,” “Anxi Tieguanyin,” “Chawentianxia” and “Huizhoulucha” contained 36.06%–38.71% neutral sugar, 31.76%–37.99% acid sugar, 4.60%–8.51% protein and 6.53%–9.65% TPP [26]. Black tea TPS (BTPS) contained protein-bound polysaccharides [27]. The MW distribution of TPS varied with teas used to prepare TPS, ranging from 9.2 kDa to 251.5 kDa for GTPS, from 5.3 kDa to 100.9 kDa for oolong tea TPS (OTPS) and from 3.8 kDa to 32.7 kDa for BTPS [28]. Based on dry tea weight, OTPS content (4.6% ± 0.2%) was higher than GTPS (4.0% ± 0.3%) and BTPS (4.2% ± 0.3%) [28]. Content of pu-erh tea TPS (PTPS) was 1.21% [29]. Crude PTPS could be separated into PTPS-1 and PTPS-2 by DEAE-52 and Sephadex G-150 column chromatography. PTPS-1 contained lower content of uronic acid, but higher contents of neutral sugar and protein than PTPS-2. The average molecular weight of PTPS-1 and PTPS-2 was 16.8 kDa and 12.1 kDa, respectively [30]. PTPS was acid heteropolysaccharide-bound with proteins and its content was increased with the extension of pu-erh tea fermentation [7].

Chemical compositions of TPS are changed with tea materials. The ratio of protein, uronic acid and neutral sugar was 32.6%:20.8%:27.3% for GTPS; 32.7%:25.4%:26.5% for OTPS; 38.0%:16.1%:18.8% for BTPS [28] and (4.2% 19.7%):(32.6%–40.4%):(15.3%–20.2%) for PTPS [7]. The molar ratio of neutral monosaccharides Rha:Ara:Xyl:Man:Gal:Glc in GTPS was 7.8:41.8:7.1:7.3:18.7:17.0. OTPS and BTPS contained no Xyl and Man and the molar ratio of neutral monosaccharides Rha:Ara:Gal:Glc was 16.2:43.7:18.0:21.9 for OTPS and 14.4:36.4:19.7:29.4 for BTPS [28]. PTPS-1 and PTPS-2 were composed of Ara, Gal, Glc, Rha, Xyl and Man with molar ratios of 24.2:23.6:5.9:3.2:1.8:1.1 and 19.3:26.9:3.2:2.7:1.3:5.5, respectively [30].

### 2.4. Effect of Preparation Methods on TPS

**Figure 1:**
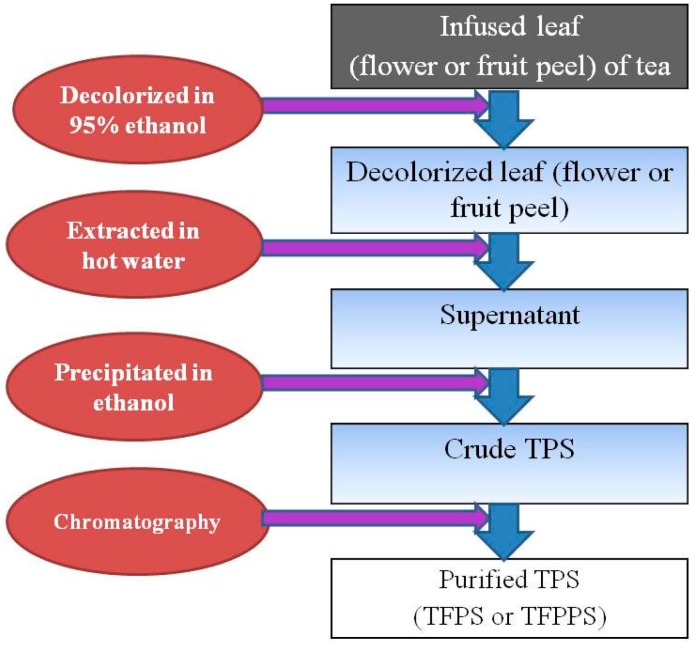
Procedure for preparing TPS.

TPS is usually extracted from tea leaves using hot water, then precipitated using ethanol of various concentrations and finally purified by chromatography (Figure 1). The optimal conditions for extracting TPS from the green tea leaf of “Anjibaicha” were 22.53 L water per kg tea leaf, extracted at 76.79 °C for 2.48 h [31]. However, the optimum conditions for extracting individual components of TPS were differentiated. Microwave heating to 170 °C was beneficial to solubilization of Ara and Gal, whereas heating above 200 °C was necessary to solubilize Xyl [32]. Enzymatic treatments will induce bioconversion of bioactive components, which can improve biological activities of TPS. Simultaneous processing with tannase and Rapidase® (DSM Gist, MA Delft, Netherlands) could improve the extraction of TPS and biotransformation of catechins with enhanced radical scavenging activity of GTPS [33]. Extrusion treatment of tea can change the monosaccharide composition, MW distribution, thermal properties and morphological properties of TPS, resulting in improvement of yield and antioxidant property of TPS. Extrusion treatment could also increase the extraction yield of TPS from 1.26% to 6.14% [34]. Supercritical CO_2_ extraction can improve the yield and bioactivity of TPS and the optimum conditions for supercritical CO_2_ extracting TPS from tea leaf were：leaf particle size 380 μm, 20% ethanol, extracting pressure 35 Mpa, extracting temperature 45 °C and extracting time 2 h, by which 92.5% of tea leaf TPS could be extracted [35]. Reverse micelle extraction technology has the advantages of high selectivity, fast mass transfer and relatively low cost, and it can be used in extraction of bioactives from plant materials. Sodium di-2-ethylhexyl sulfosuccinate (AOT) is extensively used as surfactant to form an AOT/heptane reverse micellar system in which TPS can be extracted. About 34% of forward recovery and nearly 100% of backward recovery of TPS were achieved under optimal conditions in the AOT/heptane reverse micellar system [36].

The crude TPS extracted from tea leaf using hot water could be isolated by absorbent chromatography and ion exchange chromatography into three fractions, which were heteropolysaccharide-bound with protein. The monosaccharides were differentiated between various fractions. Fraction-1 was composed of Ara, Rib, Xyl, D-glucose, Gal and Man, with MW 268 kDa and 2.8% protein; fraction-2 was composed of Ara, Rib, Glc and Man, with MW 118 kDa and 3.8% protein, while fraction-3 contained no Man, with MW 42.0 kDa and 4.0% protein. Significantly, fraction-1 showed the highest antioxidant activities [37].

The crude TPS from tea seed could also be purified by chromatography on a macroporous resin AB-8 column, in which water-soluble impurities were washed using deionized water, pigments removed using 0.25% NaOH solution, and tea seed saponin eluted using 90% ethanol. A total of 18.7 g of TPS with 89.2% purity could be isolated from 100 g tea seed [38].

Drying methods had significant influence on yield and composition of TPS. Vacuum drying gave the highest TPS yield, with 418 mg per kg green tea (418 mg/kg), and spray drying gave the lowest yield (106 mg/kg), with freeze drying (403 mg/kg) and microwave-vacuum drying (383 mg/kg) in between. However, total sugar contents were not significantly different between products obtained by various drying methods (ranging from 41.08% ± 0.799% to 42.71% ± 0.799% by dry weight). Contents of protein, TPP and Glc were the highest in TPS obtained by vacuum drying, and contents of Rha, Rib, Ara, Gal and galactose acid were the highest in TPS obtained by freeze drying, while contents of Glc, Xyl, Gal, Man, galactose acid and glucose acid were the lowest in TPS by spray drying and content of Rib was the lowest in TPS by microwave-vacuum drying [12].

Sulfation of TPS can improve hypoglycemic activity. Sulfated NTPS and ATPS could be synthesized by pyridine-sulfonic acid method [15]. Furthermore, thermal treatments, such as incubation at 98 °C for 1 h or longer, will improve the stability and antioxidant activity of ATPS [16].

## 3. Bioactivities of TPS

### 3.1. Bioavailability and Toxicity of TPS

TPS is generally recognized as a safe and non-toxic food additive. An in vitro test on dendritic cells (DCs) revealed that the cell viability showed no significant difference between TPS-treated cells at concentrations of 0.2–200 μg/mL and media-tread cells (RPMI media 1640, Gibco, BRL), during which TPS did not induce any apoptosis in DCs, showing TPS can be used for a long period without cytotoxicity [39]. An in vivo test by oral administration of TPS (5.0 g/kg BW) in mice showed that TPS had no toxicity to the liver, kidney, heart, thymus, or spleen of the tested mice and none of the mice died throughout the 15 days of experiment. There was no significant difference in the thymus index, spleen index, and liver index of the mice between the test and control groups (*p* > 0.05) [9]. Based on the Globally Harmonized System of Classification and Labeling of Chemicals (GHS) and OECD (Organization for Economic Co-operation and Development) Test Guideline 420 (fixed dose procedure), TPS was classified as GHS Category 5 [40]. Therefore, TPS can be classified as a very low toxicity substance which can thus be used for dietary supplements and as an additive in food processing [9,41,42].

There was a test showing that TPS is orally ingested and will reach the gastrointestinal tract before performing a biological function [43]. TPS with small MW is beneficial to the improvement of bioactivities [37]. TPS can form a TPS-iron complex (TPIC) when it reacts with FeCl_3_ in a weight ratio of 1:2.4 at 60 °C for 3 h. The TPIC contained 14.60% iron, and an in vitro digestion experiment on rat showed that bioavailability of TPIC was sufficient. When iron-depletion rats with blood hemoglobin as low as 90 mg/L were fed with feeds supplemented with TPIC and FeSO_4_ for 21 days, contents of hemoglobin, free erythrocyte protoporphyrin, serum iron and mean cell hemoglobin in blood of both groups of rats increased quickly to the levels of normal rats, respectively. The bioavailability of TPIC ranged from 101.85% to 116%, compared with indicator hemoglobin, serum iron and mean cell hemoglobin in rats supplemented with FeSO_4_. Therefore, the TPIC is considered a good iron supplement source for increasing uptake and bioavailability in the body [44].

### 3.2. Alleviating Oxidative Stress

TPS alleviates oxidative stress through direct scavenging of free radical species and improving activities of antioxidase enzymes. TPS is a group of heteropolysaccharides bound with proteins which can alleviate oxidative stress. The antioxidant activities of TPS vary with free radical species and molecular size of TPS. TPS showed a stronger inhibitory effect on hydroxyl radical than on superoxide radical. The 50% inhibitory concentration (IC_50_) of TPS extracted from tea leaf of “Anji Baicha” was 83.25 μg/mL on superoxide radicals and 1.69 μg/mL on hydroxyl radicals [31].When ATPS was separated by chromatography into three fractions with different molecular sizes, the fraction-3 with MW 42 kDa had stronger scavenging activity on superoxide radicals and hydroxyl radicals than the fraction-2 with MW 118 kDa and fraction-1 with MW 268 kDa [37]. The test also showed that the ability to scavenge hydroxyl radicals and superoxide radicals is related to uronic acid level in TPS. The higher the uronic acid level in TPS, the stronger its ability to scavenge hydroxyl and superoxide radicals [37]. An in vivo test in gastric cancer mice showed that TPS fraction with small MW showed stronger promoting effect on stomach antioxidant enzymes such as superoxide dismutase (SOD), catalase (CAT) and glutathione peroxidase (GSH-Px) [45]. When exhausting training mice were orally administrated by TPS (daily dosage 100–300 mg/kg BW) for 30 days, SOD, CAT, GSH-Px activities in blood, liver and heart were significantly increased, whereas malondialdehyde (MDA) level in plasma, liver and heart were reduced, compared to control mice [46].

The monosaccharide composition and molecular size range of TPS change with plant materials, resulting in differences in antioxidant activity. TPS containing Man extracted from tea leaf and tea flower had higher antioxidant activity than that extracted from tea seed [47]. Tea flower TPS (TFPS) containing a high level of sulfate and complicated monosaccharide composition had strong antioxidant activity by enhancing the activities of SOD and GSH-Px in carbon tetrachloride (CCl_4_)-induced liver injury mice and reducing the formation of MDA [48].

TPS composition varies with tea materials and places where the teas are produced, leading to difference in antioxidant activity. A test using TPS products extracted from unfermented green tea (GTPS), semi-fermented oolong tea (OTPS) and fully fermented black tea (BTPS) revealed that BTPS showed the highest antioxidant activities on hydroxyl radicals and DPPH radicals, and OTPS the least, with GTPS in between [28]. TPS extracted from green tea “Huizhoulvcha” produced in Anhui Province exhibited significantly higher superoxide anion-scavenging activity than that extracted from green tea “Xihulongjing” produced in Zhejiang Province in China [26]. Oolong tea fermentation enhanced the conjugation between TPS and protein, and so the OTPS extracted from deeply fermented oolong tea showed increased antioxidant activity [8]. However, there was a conflicting result showing that TPS extracted from non-fermented green tea had stronger scavenging activity on superoxide anion radical than that extracted from fully fermented black tea [49].

Preparation methods affect the TPS composition, resulting in differentiation in antioxidant activity. When tea fruit peel was used as material to extract tea fruit peel TPS (TFPPS), the fraction extracted in hot water contained a high level of uronic acid and showed stronger ABTS (2,2′-azinobis(3-ethylbenzothiazoline-6-sulfonic acid) diammonium salt) antioxidant activity but weaker FRAP (ferric-reducing antioxidant power) antioxidant activity than that extracted in ethanol [22]. When crude TPS was separated by stepwise ethanol precipitations, the TPS-I obtained by precipitation in 30% ethanol contained a high level of sulfuric radicals. A low level of uronic acid showed lower scavenging activities on 1,1-Diphenyl-2-picrylhydrazyl (DPPH) free radical, superoxide anion radical and hydroxyl radicals than the TPS-II prepared using the supernatant which had less sulfuric radical but higher levels of uronic acid [49]. The free radical scavenging activity of TPS was also influenced by the drying method. Freeze-dried TPS exhibited strong activity of metal chelating and superoxide radical scavenging, while vacuum-dried TPS showed high activity of inhibiting α-glycosidase and α-amylase [12].

Free radical scavenging activity of TPS depended on its concentration. DPPH radical scavenging activity of TPS increased with increasing concentration between 25 μg/mL and 200 μg/mL. When TPS concentration reached 200 μg/mL or above, the DPPH radical scavenging activity hardly changed. TPS at 25–200 μg/mL showed weaker DPPH scavenging activities than vitamin C, but exhibited similar DPPH scavenging activity with vitamin C at 200 μg/mL or above [26].

There was synergistic interaction between TPS and other bioactive tea components. Epigallocatechin gallate (EGCG) caused a synergistic increase in the antioxidant activity of TPS. Low concentration of EGCG (6.15–8.0 μg/mL) significantly enhanced DPPH radical scavenging potential and reducing power of TPS [50]. Crude TPS with low level of catechins showed stronger antioxidant activities than that of purified TPS fractions [50]. There was also synergistic interaction between TPS and polysaccharides from *Pyracantha fortuneana* (PFPS). An in vivo test on Kunming mice showed that combined oral administration of Se-enriched TPS and PFPS significantly enhanced the activities of GSH-Px and SOD, but remarkably decreased MDA level, compared to individual TPS or PFPS alone [51]. Therefore, combined administration of TPS and PFPS can synergistically improve immune function and decrease oxidative stress by enhancing the mechanisms involved in the clearance of free radicals [51].

### 3.3. Antitumor

Many in vitro tests revealed that TPS showed antitumor potential. TSPS significantly inhibited the growth of human immortalized myelogenous leukemia cell K562 at a concentration of 50 μg/mL, with an inhibition ratio 38.44% ± 2.22% (*p* < 0.01) [21]. When the TSPS was further separated into NTPS, ATPS-1 and ATPS-2, they all showed inhibitory effects on K562 cells in a dose-dependent manner, with inhibition ratios of 30.13% ± 3.54% for NTPS, 36.61% ± 2.75% for ATPS-1 and 32.33% ± 2.53% for ATPS-2 at 400 μg/mL, respectively [52]. TPS extracted from Se-enriched “Ziyang” green tea significantly inhibited the proliferation of human osteosarcoma U-2 OS cancer cells in a dose-dependent manner at 25–200 μg/mL [53]. TFPS with a high level of sulfate and complicated monosaccharide composition showed strong inhibitory activity on growth of human gastric cancer BGC-823 cells [48]. After 72 h in vitro incubation, the inhibition rates of TFPS-1 with 2.63% sulfuric radical and TFPS-3 with 1.76% sulfuric radical at a concentration of 200 μg/mL were 82.60% and 80.73%, respectively, which is significantly higher than those of crude TFPS with 1.45% sulfuric radical and TFPS-2 with 0.84% sulfuric radical [48]. An in vitro test showed that TPS (25, 50, 100 and 200 μg/mL) could significantly inhibit the proliferation of human osteosarcoma U-2 OS cells in a concentration-dependent fashion (*p* < 0.05 or *p* < 0.01) [53]. These experiments suggest that TPS will be a potential candidate for natural antitumor drugs.

The antitumor activity of TPS was also confirmed by in vivo tests. An in vivo test on U-2 OS cancer xenograft model BALB/c athymic mice showed that oral administration at three daily doses of 100, 200 and 400 mg/kg BW for 28 days resulted in obvious tumor regression as compared to model control (*p* < 0.05 or *p* < 0.01). In addition, body weights of the mice in control or TPS-treated groups did not differ significantly and no mice died during the experiment, suggesting TPS has cancer-preventive and cancer-therapeutic benefit for human osteosarcoma [53]. Oral administration of TFPS at daily dosages of 75, 150 and 300 mg/kg for 10 days inhibited the growth of transplanted sarcoma 180 tumor (S180) on S180-bearing mice, prolonged the mice survival days, promoted the plasma interleukin-2 and interferon-γ levels, and improved the T-lymphocyte subsets CD4^+^ and CD4^+^/CD8^+^ percentages [54]. In addition, TFPS was found to increase the delayed-type hypersensitivity response and macrophage phagocytosis significantly, indicating TFPS enhanced the host defense response to tumor due in part to the immunomodulatory activity [54]. TPS could inhibit the growth of H22 transplantable hepatocarcinoma (HCC) tumor in mice [55]. An in vivo test showed that TPS significantly inhibited the growth of H22 transplantable tumor in mice, remarkably decreased the spleen index and increased the thymus index compared with that of model group (*p* < 0.05). Furthermore, TPS significantly improved the splenocyte proliferation induced by concanavalin A (ConA) or lipopolysaccharide (LPS), and notably enhanced the macrophage phagocytosis towards neutral red [55]. A test on Wistar rats with H22 HCC cells confirmed that oral administration of TPS (100, 200 and 300 mg/kg BW, once a day for 40 consecutive days) inhibited tumor growth and decreased microvessel density in tumor tissue. The altered amount of serum white blood cells (WBC), interferon-gamma (IFN-γ) and tumor necrosis factor-α (TNF-α) in HCC animals were dose-dependently increased, whereas activities of serum alanine transaminase (ALT), aspartate transaminase (AST) and alkaline phosphatase (ALP) were dose-dependently decreased in the TPS-treated animals. The suppressive effect of TPS on tumor growth is also considered to be related to its inhibiting expression of vascular endothelial growth factor (VEGF) and proliferating cell nuclear antigen (PCNA) in H22 tumor tissue [56].

### 3.4. Anti-Hyperglycemia

An in vivo test showed that TPS had an inhibitory effect on blood glucose (BG) increase and diabetes mellitus (DM). When seven-week-old C57BL/8 mice were injected with TPS with MW 107 kDa–110 kDa, the BG levels in normal mice and model mice with high BG were significantly decreased by 13.54% and 22.18%, respectively [19]. Four-week oral administration of PTPS (40 mg/kg BW daily) could significantly lower the BG levels in alloxan-induced diabetic mice, accompanying improvement of activities of SOD and GSH-Px as well as MDA levels both in serum and liver [57]. Oral administration of GTPS (200 and 400 mg/kg BW daily) for six consecutive days could also suppress BG increase in alloxan-induced mice [25].

DM is an endocrine disorder caused by inherited and/or acquired deficiency in the amount of insulin from the pancreas, or by the defects in insulin action. Glucokinase is the first enzyme in glycolysis and glycogenesis; it is also a key enzyme in diabetes management, thereby serving as a signal to both the b-cells and the liver that glucose levels in the blood are high. Glucokinase plays a role in promoting insulin secretion and reducing glucose production by the liver. Glucokinase facilitates phosphorylation of glucose to glucose-6-phosphate, which is regulated by insulin. Glucokinase influences glucose uptake by liver. Increase in glucokinase activity is beneficial to alleviating the symptoms of diabetes. TPS had elements related to reducing blood sugar (ERBS), with inhibitory percentages ranging from 0.03% to 9.57% [58]. The bioactivities of OTPS were proportional to its contents of protein and uronic acid [8]. The protein and uronic acid in TPS had an inhibitory effect on α-glucosidase activities and had potential for prevention of type 2 diabetes (T2D) [10]. Pu-erh tea extracts containing TPS had beneficial effects on glucose homeostasis in T2D and in amendment of insulin resistance [29]. TPS improved impaired glucose tolerance and ameliorated retarded insulin response at 60 and 120 min in diabetic db/db mice [29]. An ATPS purified by gel filtration chromatography, which contained 8% galacturonic acid and had MW 60 kDa, showed a significantly stimulating effect on glucokinase activity, resulting in BG reduction and suppression of MD [14].

Dysfunction of the vascular endothelium contributes to the etiology of diabetic micro- and macro-angiopathy [59]. Excessive increase in intra cellular glucose induces serious loss of vascular endothelial cells [60] and accelerates the occurrence of atherosclerosis in DM patients [61]. Fractions 1–3 of GTPS obtained by extracting low-grade green tea in hot water and precipitating in ethanol, and finally fractionating on DEAE-cellulose DE-52 column showed protective effects on human umbilical vein endothelial (HUVE) cells [62]. Exposure of HUVE cells to high glucose (33 mM) for 12 h significantly decreased cell viability relative to normal glucose control (*p* < 0.001). As compared with the cell injury group, fractions 1–3 of GTPS at three dose levels (50, 150 and 300 μg/mL) showed remarkably protective effects on HUVE cells against impairments induced by high glucose in a dose-dependent manner (*p* < 0.05, *p* < 0.001). The inhibitory effects of GTPS on high glucose-mediated HUVE cell loss were, at least in part, correlated with their potential scavenging potency of reactive oxygen species (ROS) [62].

α-Amylase and α-glucosidase are key enzymes to digest starch in mammals [63]. Inhibition of starch digestive enzymes or glucose transporters can suppress postprandial hyperglycemia by reducing the rate of glucose release and absorption in the small intestine [64]. TPS improved the impaired glucose tolerance (IGT) from developing into DM through its inhibiting digestive enzymes [65]. BTPS at 25–200 μg/mL suppressed α-glucosidase activity in a concentration-dependent manner [28]. TFPS could also inhibit the activity of α-amylase and α-glucosidase in vitro. The possible mechanism for TFPS protecting against rapid BG rise in alloxan-induced Sprague-Dawley (SD) rats was that TFPS donated hydrogen to protect SD rats from oxidative damage and inhibited digestive enzyme activities [66]. PTPS decreased blood sugar by inhibiting α-glucosidase activity in vitro, with IC_50_ = 0.438–2.192 μg/mL [67].

Type 1 diabetes (T1D) is an autoimmune disorder induced by dysregulation of the immune system. During development of functional regulatory T cells (Treg), interleukin 2 (IL-2) is a necessary second signal after T cell antigen receptor (TCR), signaling that it upregulates Tregs CD25 and Foxp3. IL-2 may not only cause proliferation of Tregs, but also compensate for a genetic defect associated with T1D [68]. IL-1 has a major role in inflammation. The blockade of IL-1 activity (especially IL-1β) is a standard therapy for patients with autoimmune diseases [69]. TPS treatment promoted production of IL-2 in spleen cells but suppressed production of IL-1 in adjuvant arthritis rats in vivo [19]. The hypoglycemic mechanism of TPS is also considered to be involved in its regulation of the PI3K/Akt signal pathway because TPS was found to upregulate the expressions of PI3Kp85/p-Akt/GLUT4 in T2D mice [70]. When diabetic mice were orally gavaged with TPS dissolved in NS at the doses of 200, 400 and 800 mg/kg BW per day for 28 days, the expression of PI3Kp85, p-Akt and GLUT4 increased in a dose-dependent manner, accompanying a dose-dependent decrease in serum glucose level [70].

Anti-glutamic acid decarboxylase (anti-GAD) antibody is considered to be an important marker for T1D [71]. Daily oral administration of 150 mg/kg green tea water-soluble TPS and alkali-soluble TPS suppressed spontaneous DM in non-obese diabetic (NOD) mice by decreasing the levels of anti-GAD antibody and blood glucose [72]. The hypoglycemic activity of TPS can be further improved by molecular modification such as sulfation [15]. An in vivo test on alloxan-induced diabetic mice showed that BG levels of a sulfated NTPS group and sulfated ATPS group after administration for 7 h were 8.31 mmol/L and 8.18 mmol/L, being significantly lower than those of non-sulfated NTPS and ATPS, respectively (*p* ≤ 0.01) [15].

### 3.5. Improving Immunity

TPS can improve immunity by enhancing the activity of immunocytes such as splenocytes. Splenocytes consist of a variety of cell populations such as lymphocytes, DCs and macrophages, which have different immune functions. TPS significantly improved the splenocyte proliferation induced by ConA or LPS, and notably enhanced the macrophage phagocytosis towards neutral red [55]. TPS promoted both phenotypic and functional maturation of murine bone marrow-derived DCs, achieving potentiation of immune responses to alleviate the diseases [39]. TPS promoted the phagocytic activity of monocyte-macrophage system, resulting in enhancement of self-protection activity and increasing phagocytosis through toll-like receptor 7 [73].

Cytokines form a group of proteins with small MW released by cells that have specific effects on the interactions and communications between cells, or on the behavior of cells. The cytokines include interleukins (IL), lymphokines and cell signal molecules, such as tumor necrosis factor (TNF) and the interferons, which trigger inflammation and respond to infections. An in vivo test on Kunming mice showed that oral administration of TPS could significantly decrease the level of pro-inflammatory cytokines such as TNF-α, but could increase the level of anti-inflammatory cytokines such as serum immunoglobulin A (IgA), IgG, IgM, IL-2, IL-4, IL-10 [45] as well as IL-6 which plays an important role in T cell activation [33]. Oral administration of TFPS could also improve the percentages of T-lymphocyte subsets CD4^+^ and CD4^+^/CD8^+^ [54]. The effect of TPS on immune stimulation was superior to that of TPP to some extent [55]. Therefore, TPS can be used as an immunopotentiator.

However, the immunological activities of TPS were differentiated between various sources. The TPS from immature leaves had higher immunostimulating activity than that from mature leaves and its activities depend on the content of strictinin in the leaf extract [74]. A mixture of TPS without polyphenols and catechin did not increase the immunostimulating activity. Crude polysaccharide from tea leaf containing a lot of catechins is a potential immunostimulator, and strictinin might promote the formation of a catechin-polysaccharide complex, indicating that the catechin-polysaccharide complex is a very important molecule in the immunomodulating activity of tea extracts [74]. ATPS showed stronger immunological activity than NTPS at concentrations 0.5–400 μg/mL. The detail mechanisms of immunological activity of TPS have not been clear [52].

### 3.6. Anti-Hepatotoxicity

TPS plays a role in anti-hepatotoxicity through ameliorating hepatic oxidative injury [6] and improving metabolic syndrome [27]. Oral administration of TFPS for 28 consecutive days protected liver from lipid peroxidation induced by bromobenzene in mice through increasing SOD activity, resulting in reduction of MDA in a dose-dependent manner [75]. In vivo test on exhausting training mice showed that oral administration of TPS (100, 200 and 300 mg/kg BW) for 30 days increased the activities of SOD, catalase (CAT), GHS-Px and reduced MDA level in plasma, liver and heart [46].

Carbon tetrachloride (CCl_4_) induced hepatotoxicity, accompanying an increase in serum alanine transaminase (ALT), aspartate transaminase (AST), triglycerides (TG), cholesterol (TC), hepatic MDA and 8-iso-PGF2α (8-iso-prostaglandin F2 alpha). Administration of GTPS or BTPS (200, 400 and 800 mg/kg BW) in mice ahead of CCl_4_ injection could antagonize the CCl_4_-induced increases in levels of ALT, AST, TG, TC, hepatic MDA and 8-iso-PGF2α. The TPS-treated mice displayed a better profile of hepatosomatic index and improved GSH-Px and SOD activities. These protective effects can be attributed to TPS enhancing the effects on enzymatic and non-enzymatic antioxidants and restraining lipid peroxidation in liver tissue [27,48,76].

Nitric oxide (NO) is a free radical which can be produced by nitric oxide synthase (NOS) in the body. There are three NOS isoforms identified in the body, i.e., endothelial nitric oxide synthase (eNOS), neural nitric oxide synthase (nNOS) and inducible nitric oxide synthase (iNOS). The iNOS is inducible in response to various stimuli, such as LPS which can activate Toll-like receptor 4 (TLR4) signal pathway [77]. Tests showed that PTPS suppressed the increase in level of LPS-induced NO in SD rats by inhibiting iNOS expression through reducing TLR4 signaling [78]. The SD rats fed with PTPS extracted from pu-erh tea at a daily dose 50 mg/kg BW for four weeks had less expression of iNOS mRNA. The relative mRNA unit of PTPS groups was 48% of that in control group (water + LPS) [78].

### 3.7. Anti-Skin Aging

An in vitro test on senescent human diploid fibroblast (HDF) showed that PTPS promoted proliferation of HDF significantly and the anti-aging effect of TPS on HDF was even stronger than vitamin C and TPP [79]. The abilities of TPS and TPP to protect the skin were assessed from four aspects, i.e., moisture absorption and retention, sunscreen, promoting the proliferation of fibroblast cells, and tyrosinase inhibitory ability. Purified TPS had better moisture absorption and retention abilities than TPP. TPP protected skin against the sun’s ultraviolet (UV) radiation, enhanced proliferation of fibroblast cells and had an inhibitory effect on tyrosinase, whereas purified TPS hardly protected the skin from UV rays and showed weak ability to inhibit tyrosinase. TPS and TPP had complementary advantages and they should be appropriately combined to achieve higher performance when applied as active components in cosmetics [80]. A six-month double-blind, placebo controlled, randomized study on healthy post-menopausal females showed that a dietary supplement containing white tea extract and fish protein polysaccharides provided improved condition, structure and firmness of the skin in post-menopausal women, showing improvement of forehead, periocular and perioral wrinkles, mottled pigmentation, laxity, sagging, under eye dark circles and overall appearance [81].

### 3.8. Anti-Infection of Pathogenic Bacteria

The adhesion of the pathogen to host cells is the first step during bacterial infection. Anti-adhesion therapy is an efficient way to prevent or treat bacterial infections. TPS showed selectively strong inhibition on bacteria-host adhesion. ATPS with a MW 80 kDa showed marked anti-adhesive effects against pathogenic bacteria such as *Helicobacter pylori, Propionibacterium acnes*, and *Staphylococcus aureus* with a minimum inhibitory concentration (MIC) between 0.01 and 0.1 mg/mL, which was lower than polysaccharides extracted from *Panax ginseng* and *Artemisia capillaries* [82]. A TPS-like green tea extract containing 40% uronic acid, but lack of catechins, showed strong inhibitory effects on the adhesion of some pathogens to host cells, with IC_50_ (50% inhibition of adhesion) values being 0.14–2.30 mg/mL for pathogens *H. pylori, P. acnes* and *S. aureus.* The inhibitory effects of TPS depend on the pathogen species and it exhibited the highest activity against *P. acnes*, but no inhibition against *Lactobacillus acidophilus, Bifidobacterium bifidum, Escherichia coli,* or *Staphylococcus epidermidis*, suggesting TPS exerted a selective anti-adhesive effect against certain pathogenic bacteria with no adverse effects against some commensal bacteria [83].

The detailed mechanisms for TPS interfering with bacteria-host adhesion remain to be investigated. The negatively charged groups on TPS molecules may perform a crucial role in the process of bacteria-host adhesion. TPS and pectin have a similar carbohydrate composition, in which uronic acids are abundant. However, pectin alone did not show any significant inhibition effect on the bacteria-host adhesion in a concentration-dependent manner. Some carbohydrate components of TPS other than uronic acid might play a role in the observed inhibition of host-bacterial adhesion [84], which remains to be further investigated.

## 4. Inconsistent Results

Although in vitro and in vivo tests showed that TPS exhibited many bioactivities, there were also inconsistent or controversial results published in this research area.

### 4.1. Inconsistent Chemical Composition of TPS

Crude TPS could be separated into two fractions [13], three fractions [37] or five fractions [14] according to the isolation method and materials used, resulting in variations in chemical composition and bioactivities. The content of the major component, uronic acid, in TPS varied from 1.95%–7.90% [49] to 45.89%–63.11% [8]. The content of protein in TPS changed from 1.5%–2.9% [85] to 32.6%–38.0% [28]. TPS MW distributed from 1−800 kDa [26] to 10−640 kDa [8]. Because the composition varied greatly, the extraction yield of TPS changed vastly from 0.23%–0.58% [19] to 4.0%–4.6% by dry weight [28] (Table 1).

### 4.2. Controversial Antioxidant Activities

Crude TPS usually contained TPP and so showed good antioxidant activities [12]. However, purified TPS fractions free from TPP hardly exhibited antioxidant activities, which were similar to that of dextrans. TPS as food antioxidant was considered to be an old wives’ tale [86]. Furthermore, inconsistent results of antioxidant activities of TPS came from experiments on TPS extracted using different kinds of teas with various degrees of fermentation. The early test showed that BTPS from fully fermented black tea had the highest antioxidant activities on both hydroxyl radicals and DPPH radicals, whereas OTPS from semi-fermented oolong tea had the least, with GTPS from unfermented green tea in between [28]. Fermentation of oolong tea increased the conjugation between TPS and protein, leading to increased antioxidant activity [8]. However, the later experiment showed that TPS from less intensively fermented tea such as green tea had higher antioxidant activity than those from more deeply fermented teas [49].

### 4.3. Inconsistent Relationship between Single Bioactive Compound Content and Bioactivity

Uronic acid residue can alter properties of polysaccharides and modify their solubility, and carboxyl groups of the uronic acid might play a role in hydrogen-donating and electron-transferring. TPS containing high levels of uronic acid was considered to have high biological effects [49,85,87]. However, TFPPS-60, which was extracted and purified from tea fruit peel and had 46.42% uronic acid, showed stronger ability on ferric-reducing antioxidant power (FRAP) than crude TFPPS with 68.96% uronic acid. The strong reducing ability of TFPPS-60 might be attributed to its high content of neutral sugar [22]. Though crude TFPS which was extracted from tea flower and had 22.75% uronic acid and 1.45% sulfuric radical showed stronger scavenging activities on superoxide anion radical and DPPH radical than purified TFPS-1 with 1.40% uronic acid and 2.63% sulfuric radical, TFPS-1 showed stronger inhibitory effects against human gastric cancer BGC-823 cells than crude TFPS [48]. Man was considered to be important contributor to TPS antioxidant activity. TLPS and TFPS containing Man had higher antioxidant activity than TSPF without Man [47]. However, BTPS without Man showed lower DPPH radicals IC_50_ (20.3 ± 2.6 μg/mL) and hydroxyl radicals IC_50_ (352.3 ± 12.0 μg/mL) than GTPS with 7.3% Man (DPPH radicals IC_50_ 23.0 ± 2.9 μg/mL and hydroxyl radicals IC_50_ 424.3 ± 13.6 μg/mL) [28]. These suggest that it is difficult to predict the bioactive potential using single compound indicator.

### 4.4. Inconsistent Relationship between TPS Molecular Weight (MW) and Bioactivity

Molecular size of TPS was once considered to be an important parameter affecting antioxidant activity of TPS. TPS fractions with lower MW were found to show higher antioxidant activities than those with higher MW [28,37,85]. The relationship of molecular weight to antioxidant activity of TPS was not confirmed in many other experiments [8,12,34]. The IC_50_ of TPSE12 with MW 4–05 kDa on DPPH radical scavenging activity was lower than TPSE4 with MW 15–30 kDa (IC _50_ = 4.66 mg/mL) and TPSU with MW 1–30 kDa (IC_50_ = 5.25 mg/mL) though they were all extracted from coarse green tea leaves [34].

### 4.5. Further Study Suggestions

There was a causal relationship between the unstable chemical composition and the inconsistent results of antioxidant activities of TPS, in which the former might be the cause and the latter the consequence. Purified TPS without contaminants should be obtained before it can be used in a validation test or as functional food additives. Differences in preparation methods and tea materials are important factors leading to variation in chemical composition of final TPS products. It is necessary to establish a set of effectively standardized methods to purify TPS for scientific research and industrial use in medicinal and functional food areas.

## 5. Conclusions

Tea polysaccharides (TPS) comprises a group of bioactive components in tea. Crude TPS was usually prepared by extracting tea leaf (or flower, fruit peel) in hotnwater and then precipitating in ethanol solution at different concentrations. The crude TPS could be further purified by chromatography, such as gel filtration, ion-exchange, or affinity chromatography. TPS is mostly glycoconjugates in which a protein carries one or more carbohydrate chain covalently attached to a polypeptide backbone. TPS is also typically composed of heteropolysaccharides in which uronic acids are abundant.

**Figure 2:**
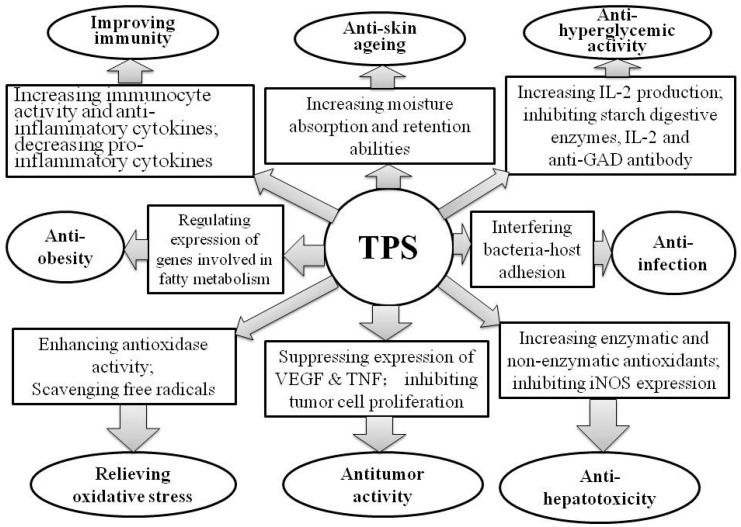
Bioactivities of TPS.

TPS has many bioactive activities, including relieving oxidative stress by enhancing endogenous antioxidant enzymes or directly scavenging free radicals; antitumor activity by suppressing the expression of VEGF and TNF and inhibiting tumor cell proliferation; anti-hyperglycemic activity by increasing IL-2 production and inhibiting starch digestive enzymes, IL-2 and anti-GAD antibody; improving immune activity by enhancing immunocyte activity, increasing the level of anti-inflammatory cytokines such as IgA, IgG, IgM, IL-2, IL-4, IL-10 but decreasing pro-inflammatory cytokines such as TNF-α AND IL-6; anti-hepatotoxicity by increasing enzymatic and non-enzymatic antioxidants and inhibiting iNOS expression via reducing TLR4 signaling; anti-skin-aging by increasing moisture absorption and retention abilities; and anti-infection of bacteria by interfering bacteria-host adhesion. Furthermore, TPS plays a role in weight control by downregulating the genes related to fatty metabolism, such as gene Lpin2 in the pathway of triacylglycerol biosynthesis [88] (Figure 2).

Differences in preparation methods and raw tea materials are considered to be important factors leading to variation in chemical composition and antioxidant activities of TPS (Table 1). A set of efficient and standardized methods to purify TPS from various kinds of tea should be established so as to obtain purified TPS products with stabilized chemical compositions for validation test or use as medicinal and food additives.

## Figures and Tables

**Table 1:** Effects of preparation methods and raw materials on chemical composition of TPS.

Preparation Method	Raw Materials	Major Components	MW Distribution	Reference
Separated by DEAE-cellulose column and dried by freeze drying	Green tea	TPS was composed of Rha, Rib, Ara, Xyl, Man, Glu, Gal in molar contents of 53.92, 13.07, 94.56, 38.61, 24.75, 85.38, 124.50 mM.	TPS had a largely undispersed MW distribution.	Guo et al., 2011 [4].
Extracted in hot water and precipitated in ethanol, then lyophilized	Oolong teas Tieguanyin (TTPS), Fenghuangdancong (FTPS) and Dahongpao (DTPS)	TTPS contained 5.57% protein, 27.56% neutral sugars and 45.89% uronic acid; FTPS contained 7.68% protein, 16.71% neutral sugars and 56.46% uronic acid; DTPS contained 9.30% protein, 20.74% neutral sugars and 63.11% uronic acid	7–817 kDa for TTPS, 14–930 kDa for FTPS and 42–264 kDa for DTPS	Wang et al., 2012 [8]
RP-C18 column chromatography	Green tea	Man, Rib, Rha, GulA, GalA, Glu, Xyl, Gal And Ara in molar contents of 16.3, 10.3, 47.1, 5.6, 24.0, 128.4, 25.0, 101.4 and 71.1 μM		Lv et al., 2009 [10]
Extracted in hot water and precipitated in ethanol, then dried by freeze drying (TPS-F), spray drying (TPS-S), vacuum drying (TPS-V) and microwave-vacuum drying (TPS-M)	Green tea	Total sugar content was 42.71% in TPS-F, 42.01% in TPS-V, 41.16% in TPS-S and 41.08% in TPS-M. Protein content was 5.5% in TPS-F, 8.34% in TPS-V, 4.10% in TPS-S and 5.75% in TPS-M. Polyphenols content was 10.41% in TPS-F, 13.22% in TPS-V, 9.56% in TPS-S and 10.78% in TPS-M	3.3–952.5 kDa for TPS-F, 3.4–910.9 kDa for TPS-V, 3.3–969.1 kDa for TPS-S and 3.5–915.7 kDa for TPS-M	Wang et al., 2013 [12]
Hot water extraction and followed by 40% (TPS1) and 70% (TPS2) ethanol precipitation	Green tea	TPS1 showed stronger phagocytosis-enhancing activity than TPS2.	TPS1-2a had MW 20 kDa and TPS1-2b had a MW 22 kDa.	Wang et al., 2014 [13].
Anion-exchange chromatography	Green tea,	Water-soluble TPS was separated into 5 fractions among which fraction C was further separated into FC-1 and FC-2. FC-1 is a novel polysaccharide which is composed of Rha, Ara, Man, Glc and Gal in the ratio of 12.57:22.95:4.4:39.34:20.77, with 8% GalA.	FC-1 has a MW about 60 kDa.	Wang et al., 2006 [14]
Extraction in hot water and precipitated using 95% ethanol	Green tea	TPS was composed of Ara, Xyl, Fuc, Glc, and Gal, with extraction yield 2.3–5.8 g per kg dry tea.	107–110 kDa	Wang et al., 2001 [19]
Hot water extraction and followed by 95% ethanol precipitation	Green tea (GTPS), Oolong tea (OTPS), Black tea (BTPS)	Protein content was 32.6% in GTPS, 32.7% in OTPS and 38.0% in BTPS. Uronic acid content was 20.8% in GTPS, 25.5% in OTPS and 16.1% in BTPS. Neutral sugar content was 27.3% in GTPS, 26.5% in OTPS and 18.8% in BTPS. Extraction yield was 4.0% for GTPS, 4.6% for OTPS and 4.2% for BTPS.	9.2–251.5 KDa for GTPS ; 5.3–100.9 kDa for OTPS and 3.8–32.7 KDa for BTPS	Chen et al., 2009 [28]
Absorbent chromatography and ion exchange chromatography	Green tea	TPS-1 was composed of Ara, Rib, Xyl, Glc, Gal and Man, with 30.0% uronic acid and 2.8% protein. TPS-2 was composed of Ara, Rib, Xyl, Glc and Man with 47.6% uronic acid and 3.8% protein. TPS-3 was composed of Ara, Rib, Xyl, Glc and Gal with 51.8% uronic acid and 4.0% protein.	268 kDa for TPS-1, 118 kDa for TPS-2 and 42 kDa for TPS-3.	Chen et al. 2008 [37]
Extraction in hot water and precipitated in 30%–60% final ethanol	Green tea (GTPS), Dark tea (DTPS), Oolong tea (OTPS), White tea (WTPS) and Black tea (BTPS)	Sulfuric radical content was 0.37%–0.91% in GTPS, 0.55%–1.78% in DTPS, 2.77%–3.44% in OTPS, 0.99%–1.21% in WTPS and 1.65%–2.13% in BTPS. Uronic acid content was 3.40%–2.18% in GTPS, 9.90%–6.05% in DTPS, 6.42%–3.71% in OTPS, 56.51%–2.03% in WTPS and 3.21%–1.95% in BTPS		Zhao et al., 2014 [49]
Extraction in hot water and precipitated using 95% ethanol	Tea flower (TFPS)	TFPS-1 was composed of Glc:Xyl:Rha:Gal in ratio of 1.0:1.2:0.81:0.98; TFPS-2 comprised Glc:Xyl:Rha:Ara in ratio of 1.0:0.76:2.3:2.3.	167.5 kDa for TFPS-1 and 10.1 kDa for TFPS-2	Han et al., 2011 [85]

## Abbreviations

**Abbreviation**	**Full Name**	**Abbreviation**	**Full Name**
ABTS	2,2′-Azinobis(3-ethylbenzothiazoline-6-sulfonic acid) diammonium salt	IGT	Impaired glucose tolerance
ALP	Alkaline phosphatase	IL	Interleukin
ALT	Alanine transaminase	iNOS	Inducible nitric oxide synthase
AOT	Sodium di-2-ethylhexyl sulfosuccinate	LPS	Lipopolysaccharide
Ara	Arabinose	Man	Mannose
AST	Aspartate transaminase	MDA	Malondialdehyde
ATPS	Acid tea polysaccharides	MIC	Minimum inhibitory concentration
ATSPS	Acid tea seed polysaccharides	MW	Molecular weight
BG	Blood glucose	nNOS	Neural nitric oxide synthase
BTPS	Black tea polysaccharides	NOD	Non-obese diabetic
BW	Body weight	NOS	Nitric oxide synthase
CAT	Catalase	NTPS	Neutral tea polysaccharides
CCl_4_	Carbon tetrachloride	OECD	Organization for Economic Co-operation and Development
ConA	Concanavalin A	OTPS	Oolong tea polysacharides
DC	Dendritic cell	PCNA	Proliferating cell nuclear antigen
DM	Diabetes mellitus	PFPS	*Pyracantha fortuneana* Polysaccharides
DPPH	1,1-Diphenyl-2-picrylhydrazyl	PTPS	Pu-erh tea polysaccharides
EGCG	Epigallocatechin gallate	REE	Rare earth elements
eNOS	Endothelial nitric oxide synthase	Rha	Rhamnose
ERBS	elements related to reducing blood sugar	Rib	Ribose
FRAP	Ferric-reducing antioxidant power	SOD	Superoxide dismutase
Fuc	Fucose	T1D	Type 1 diabetes
GAD	Glutamic acid decarboxylase	TC	Cholesterol
Gal	Galactose	TCR	T cell antigen receptor
GalA	Galacturonic acid	TFPPS	Tea fruit peel polysaccharides
GHS	Globally Harmonized System	TFPS	Tea flower polysaccharides
Glc	Glucose	TG	Triglycerides
GSH-Px	Glutathione peroxidase	TLPS	Tea leaf polysaccharides
GTPS	Green tea polysacharides	TLR4	Toll-like receptor 4
GulA	Glucuronic acid	TNF-α	Tumor necrosis factor-alpha
HCC	Hepatocarcinoma	TPS	Tea polysaccharides
HDF	Human diploid fibroblast	TPSIC	TPS-iron complex
HG	Homogalacturonan	Treg	Regulatory T cell
HMPS	Hawk mature tea polysaccharides	TSPS	Tea seed polysaccharides
HUVE	Human umbilical vein endothelial	TPP	Tea polyphenols
IC50	50% Inhibitory concentration	UV	Ultraviolet
8-iso-PGF2α	8-Iso-prostaglandin F2 alpha	VEGF	Vascular endothelial growth factor
IFN-γ	Interferon-gamma	WBC	White blood cells
Ig	Immunoglobulin	Xyl	Xylose
